# A yeast tRNA mutant that causes pseudohyphal growth exhibits reduced rates of CAG codon translation

**DOI:** 10.1111/mmi.12096

**Published:** 2012-12-04

**Authors:** Alain J Kemp, Russell Betney, Luca Ciandrini, Alexandra C M Schwenger, M Carmen Romano, Ian Stansfield

**Affiliations:** 1 Institute of Medical Sciences, University of AberdeenAberdeen, AB25 2ZD, UK; 2 Institute of Complex Systems and Mathematical Biology, King's College, University of AberdeenAberdeen, AB24 3UE, UK

## Abstract

In *Saccharomyces cerevisiae*, the *SUP70* gene encodes the CAG-decoding tRNA^Gln^_CUG_. A mutant allele, *sup70-65,* induces pseudohyphal growth on rich medium, an inappropriate nitrogen starvation response. This mutant tRNA is also a UAG nonsense suppressor via first base wobble. To investigate the basis of the pseudohyphal phenotype, 10 novel *sup70* UAG suppressor alleles were identified, defining positions in the tRNA^Gln^_CUG_ anticodon stem that restrict first base wobble. However, none conferred pseudohyphal growth, showing altered CUG anticodon presentation cannot itself induce pseudohyphal growth. Northern blot analysis revealed the *sup70-65* tRNA^Gln^_CUG_ is unstable, inefficiently charged, and 80% reduced in its effective concentration. A stochastic model simulation of translation predicted compromised expression of CAG-rich ORFs in the tRNA^Gln^_CUG_-depleted *sup70-65* mutant. This prediction was validated by demonstrating that luciferase expression in the mutant was 60% reduced by introducing multiple tandem CAG (but not CAA) codons into this ORF. In addition, the *sup70-65* pseudohyphal phenotype was partly complemented by overexpressing CAA-decoding tRNA^Gln^_UUG_, an inefficient wobble-decoder of CAG. We thus show that introducing codons decoded by a rare tRNA near the 5′ end of an ORF can reduce eukaryote translational expression, and that the mutant tRNA_CUG_^Gln^ constitutive pseudohyphal differentiation phenotype correlates strongly with reduced CAG decoding efficiency.

## Introduction

Organisms respond to changes in environment through controlling patterns of gene expression. Gene regulation is frequently exerted at the level of transcription, although it is well understood that translational control, as well as regulation of mRNA and protein stability, also play important roles in setting the steady-state level of protein expression for any given gene. Many examples of translational control operate by controlling the ability of an mRNA to recruit ribosomal subunits during the translation initiation process. In both prokaryotes and eukaryotes, secondary structure elements within the 5′ untranslated region (5′UTR), often subject to specific binding by RNA-binding proteins, regulate ribosome access to the AUG codon and subsequently, the open reading frame (Ikemura, 1982; Sharp and Li, 1987; Kuhn and Hentze, 1992; Klausner *et al*., 1993; Vega Laso *et al*., 1993; Dong *et al*., 1996; Grunberg-Manago, 1999).

There are however a growing number of examples where the translational efficiency of an mRNA is regulated at the level of translation elongation, particularly in organisms where there is biased codon composition within genes (Sharp and Li, 1987). Biased codon usage is matched by corresponding bias within the decoding tRNA population (Ikemura, 1982; Dong *et al*., 1996), thus rare codons are decoded slowly by cognate, low abundance tRNAs. Such slow decoding may cause ribosomal queuing, which in turn can extend back to the 5′ end of an mRNA and impact upon ribosome recruitment at the initiation level. Alternatively, slow decoding of a rare codon could destabilize the parent mRNA through *ssrA*-mediated turnover in bacteria (Keiler *et al*., 1996) or via no-go decay in eukaryotes (Doma and Parker, 2006) reviewed in Buchan and Stansfield (2007). Evidence for rare codon regulation of gene expression comes from studies in a range of systems; replacing multiple rare codons with synonymous but frequently used counterparts in the *Escherichia coli* chloramphenicol acetyltransferase gene increases its expression levels (Komar *et al*., 1999), while the sequential introduction of rare AGG codons into an open reading frame proportionately reduces expression of the encoded protein (Rosenberg *et al*., 1993). These observations are supported by modelling of translation that indicate that rare codon placement in open reading frames can be highly influential in regulating protein productivity from a given mRNA (Tuller *et al*., 2010).

Other, natural examples of regulation via translation elongation are also known. The *bldA* mutants of *Streptomyces coelicolor* cannot form aerial mycelia during late stages of growth, and do not produce the expected growth stage-specific antibiotics (Merrick, 1976). *bldA* encodes the developmentally regulated rare leucine-decoding tRNA_UAA_ whose cognate TTA codon is largely absent from genes expressed during exponential growth, but is present in *adpA*, the master regulator of mycelial production (Leskiw *et al*., 1991; Li *et al*., 2007). Replacement of TTA by the more common TTG cognate leucine codon restores high level *adpA* expression (Takano *et al*., 2003). The *Streptomyces* switch to secondary metabolism and aerial mycelium production is thus controlled by the developmental regulation of *bldA* tRNA.

In *Saccharomyces cerevisiae*, several tRNA mutants are known that display *bldA*-analogous developmental defects. Mutations in the *S. cerevisiae* single-copy *SUP70* gene, encoding the glutamine-decoding tRNA^Gln^_CUG_, generate a constitutive pseudohyphal growth phenotype (Murray *et al*., 1998). Whereas diploid wild-type yeast bud in a bipolar manner to produce separate ellipsoid cells, nitrogen starvation triggers cell elongation, and unipolar budding to produce long filamentous chains known as pseudohyphae (Gimeno *et al*., 1992). However, diploid *sup70-65* mutants undergo pseudohyphal growth even when grown on media containing an abundant source of nitrogen. It was hypothesized that the tRNA^Gln^_CUG_ mutations somehow impair the sensing of the cell nitrogen supply, although there is evidence this is not via the mitogen-activated protein kinase (MAPK) cascade or the cyclic AMP-dependent Protein Kinase A (PKA) pathway known to signal pseudohyphal growth (Pan and Heitman, 1999) (Murray *et al*., 1998). Thus the mechanistic basis of how *SUP70* tRNA mutations trigger pseudohyphal growth is unclear.

In order to address the mechanistic basis of the tRNA^Gln^_CUG_ pseudohyphal growth phenotype it is necessary to identify the mechanism by which the *sup70* tRNA mutations signal to nitrogen sensing machinery. Previous studies of the *sup70* alleles, including measured expression levels of CAG codon-enriched β-galactosidase reporter genes, indicated that the mutants were probably not compromised in their ability to translate CAG codons (Murray *et al*., 1998). However, we have now re-investigated this in much more detail using a broader range of methods, and discovered that the *sup70-65* tRNA is in fact inefficiently charged with glutamine, and furthermore is unstable, leading to a large reduction in the global capacity to decode CAG during translation. We furthermore show that introduction of additional CAG codons at the 5′ end of an ORF significantly compromises reporter expression in a *sup70* mutant, revealing a clear signature of translational elongation defects during CAG decoding in this genetic background. The work thus establishes the clear principle that altering codon decoding rates during eukaryote translation elongation can significantly impact on gene expression, probably through the establishment of ribosomal queues that modulate ribosome recruitment. The study establishes the further principle that altering the translational decoding rate of the CAG codon generates a highly specific pseudohyphal growth phenotype in yeast.

## Results

### Yeast *SUP70* tRNA gene mutations cause pseudohyphal growth in N-replete liquid medium

Provided with sufficient nitrogen in the growth medium, diploid *S. cerevisiae* grows as ellipsoid cells that bud in a bipolar pattern to form round, smooth colonies on solid agar medium. Under limiting nitrogen conditions on solid medium, diploid *S. cerevisiae* with a Σ1278b genetic background will switch to pseudohyphal growth, budding in a unipolar manner to produce chains of elongated cells that radiate from the colony circumference to give the margins of their colonies a ruffled appearance (Gimeno *et al*., 1992). It has been previously reported that control over pseudohyphal growth is however lost in strains carrying specific mutations in the *SUP70* gene encoding tRNA^Gln^_CUG_. The *sup70-65* and *sup70-33* alleles trigger pseudohyphal growth on both nitrogen-limited and nitrogen-replete solid medium (Murray *et al*., 1998). In the first instance, we sought to further characterize this phenotype by analysing the behaviour of strains carrying these alleles on solid and in liquid medium.

**Fig. 1:**
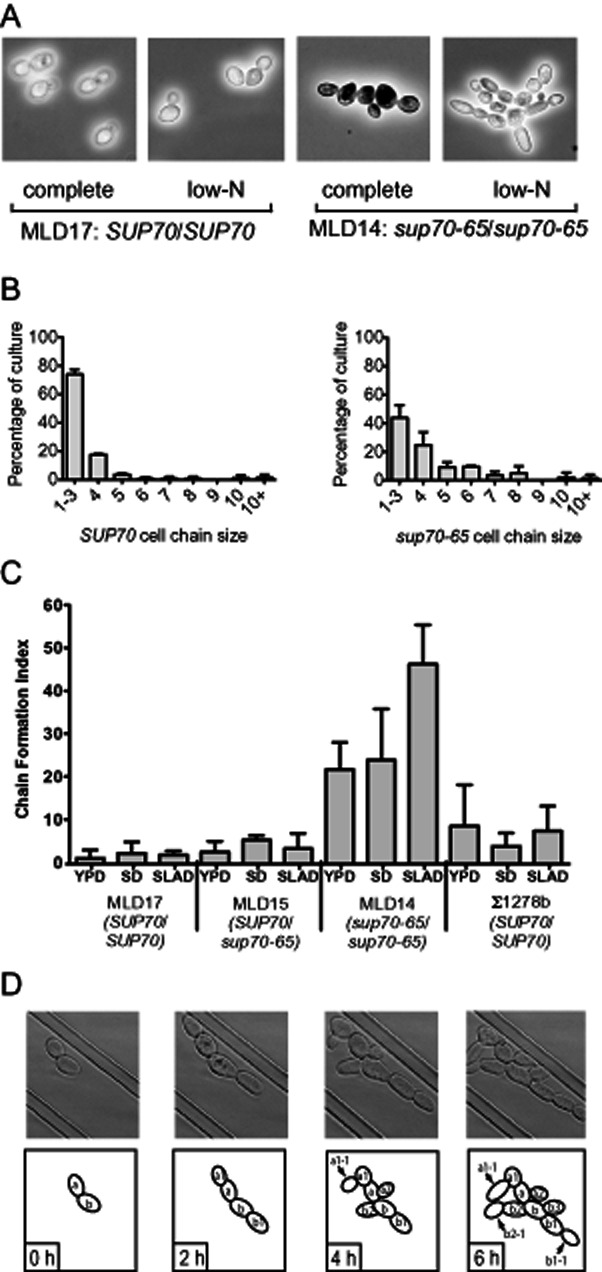
Mutations in the yeast tRNA^Gln^_CUG_ *SUP70* gene trigger pseudohyphal growth in liquid growth medium. A. *sup70-65/sup70-65* diploid mutants (MLD14), but not wild-type (MLD17) cells, form pseudohyphal chains in both liquid YPD complete medium and liquid SLAD nitrogen-depleted medium (low-N). B. Quantification of pseudohyphal chain formation in wild-type (MLD17) and *sup70-65/sup70-65* (MLD14) YPD cultures (error bars show ± 1 standard deviation, *n* = 3). C. Quantification of the degree of pseudohyphal chain formation using the chain formation index (CFI: see *Experimental procedures*). Wild-type (MLD17) and *sup70-65/sup70-65* (MLD14) strains were grown in N-replete (YPD), synthetic defined (SD) and N-depleted media (SLAD). Results are derived from counting 200–300 cells in three independent cultures. Error bars represent ± 1 standard deviation. D. Two-hour interval live-cell imaging of pseudohyphal growth of *sup70-65/sup70-65* diploid cells, with the diagrammatic lineage below indicating alternating bipolar budding of elongated cells.

Accordingly, diploid strain MLD14 (*sup70-65/sup70-65*) and the corresponding wild-type MLD17 (both generous gifts from Prof. R.A. Singer) were grown on either solid rich (YPD) or minimal media (SLAD), the latter containing limiting ammonium sulphate and known to trigger pseudohyphal differentiation in competent yeast strains (Gimeno *et al*., 1992). However, we could find no evidence of pseudohyphal differentiation on agar. All colonies were smooth-edged, lacking chains of cells at the circumference, in contrast to the phenotype reported for another *sup70-65* homozygous diploid, LMD651U (Murray *et al*., 1998). However, in this study, when the MLD diploids were tested in the corresponding liquid media, the *sup70-65* strain, but not the wild-type, underwent clear and marked pseudohyphal-type differentiation. The mutant grew as chains of ovoid cells, varying in length between four and more than 10 cells per chain in both N-replete and N-limiting medium (Fig. 1A and B). In order to quantify this phenotype, we calculated a cumulative total for cell chains, and from this, a chain formation index (CFI) was derived (Fig. 1C: *Experimental procedures*) which allowed us to quantitatively compare chain formation under different conditions. Using this index, it was apparent that the degree of pseudohyphal-type differentiation in the mutant was significantly greater in N-limiting SLAD medium than that measured in SD or YPD media (Fig. 1C).

To further characterize the chain formation phenotype, we used live microscope observation of MLD14 growth in liquid SLAD medium over a 6 h period (Fig. 1D). Unexpectedly, the budding pattern was bipolar, and not unipolar as described for pseudohyphal growth (Gimeno *et al*., 1992). The daughter cells generally remained attached to the mother, accounting for the clustering, and were significantly elongated, with an axial ratio of 1.7, compared with the wild-type value of 1.5. The *sup70-65* homozygote MLD14 thus exhibited an atypical pseudohyphal growth phenotype, revealed only in liquid medium.

**Fig. 2:**
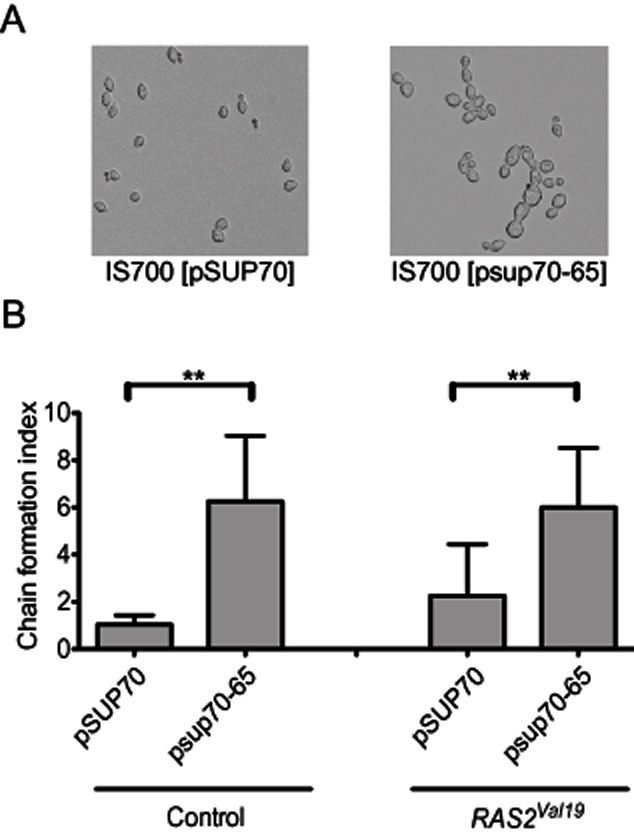
Homozygous *sup70-65* tRNA mutations trigger pseudohyphal growth in S288C-background yeast strains. A. Micrographs of the BY4743-derived diploid strain IS700 (Δ*SUP70/*Δ*SUP70*) supported with a plasmid-borne copy of either *SUP70* or *sup70-65*, revealing that the latter transformants exhibit clear pseudohyphal formation in SD liquid medium, despite the presence of a homozygous *flo8-1* mutation derived from the S288C parental background. B. Pseudohyphal chain formation was quantified (chain formation index), in strain IS700 Δ*SUP70/*Δ*SUP70* transformed with either a plasmid expressing wild-type (pSUP70) or mutant (psup70-65) alleles of the tRNA^Gln^_CUG_ gene. Strains were additionally transformed with either a control vector or one expressing a dominant *RAS2^Val19^* allele, known to stimulate pseudohyphal growth in the Σ1278b genetic background. Chain formation was assayed in triplicate SD cultures (200–300 cells counted/culture). Error bars represent ± 1 standard deviation (*n* = 3). Significance was calculated using a Student's *t*-test (*P = 0.05, **P = 0.025, ***P ≤ 0.005).

The MLD strains that were tested above were derived from the Σ1278b background, known to form pseudohyphae. As a further test of the *sup70-65* phenotype, we asked whether this tRNA mutant could trigger pseudohyphal-type growth in an S288C-background diploid, which carries a *flo8-1* mutation preventing archetypal pseudohyphal growth (Liu *et al*., 1996). We therefore created a homozygous *SUP70* knockout in the sequenced diploid strain BY4743, complemented with a plasmid-based copy of either *SUP70* or *sup70-65* (Fig. 2). While the *SUP70* transformant presented a wild-type phenotype, surprisingly, complementation of the *sup70* homozygous deletion with a plasmid-borne *sup70-65* allele successfully induced a chain formation phenotype, albeit less pronounced than that measured in the MLD strain background (Σ1278b-derived). The detection of chain formation in the S288C-derived strain was despite the absence of functional Flo8p, another indication that the *sup70-65* pseudohyphal growth form was atypical. Both strains were then transformed with an additional plasmid-borne copy of constitutively active *RAS2*^Val19^, a dominant mutation that constitutively activates the yeast RAS-cAMP pathway (Toda *et al*., 1985) and enhances pseudohyphal growth (Gimeno *et al*., 1992; Lorenz and Heitman, 1997) (Fig. 2B). However the degree of chain formation was not significantly enhanced by the mutant *RAS2*^Val19^ allele in either wild-type or *sup70-65* mutant. Although it is formally possible that the Ras2 pathway is maximally activated in these strains, we consider it more likely that the enhanced Ras2 pathway signalling achieved using the Val19 mutant was not capable of inducing an additional liquid medium chain formation response, suggesting that this liquid medium chain phenotype was not being signalled via the RAS-cAMP pathway.

### Novel tRNA mutants capable of nonsense suppression do not cause pseudohyphal growth

**Fig. 3:**
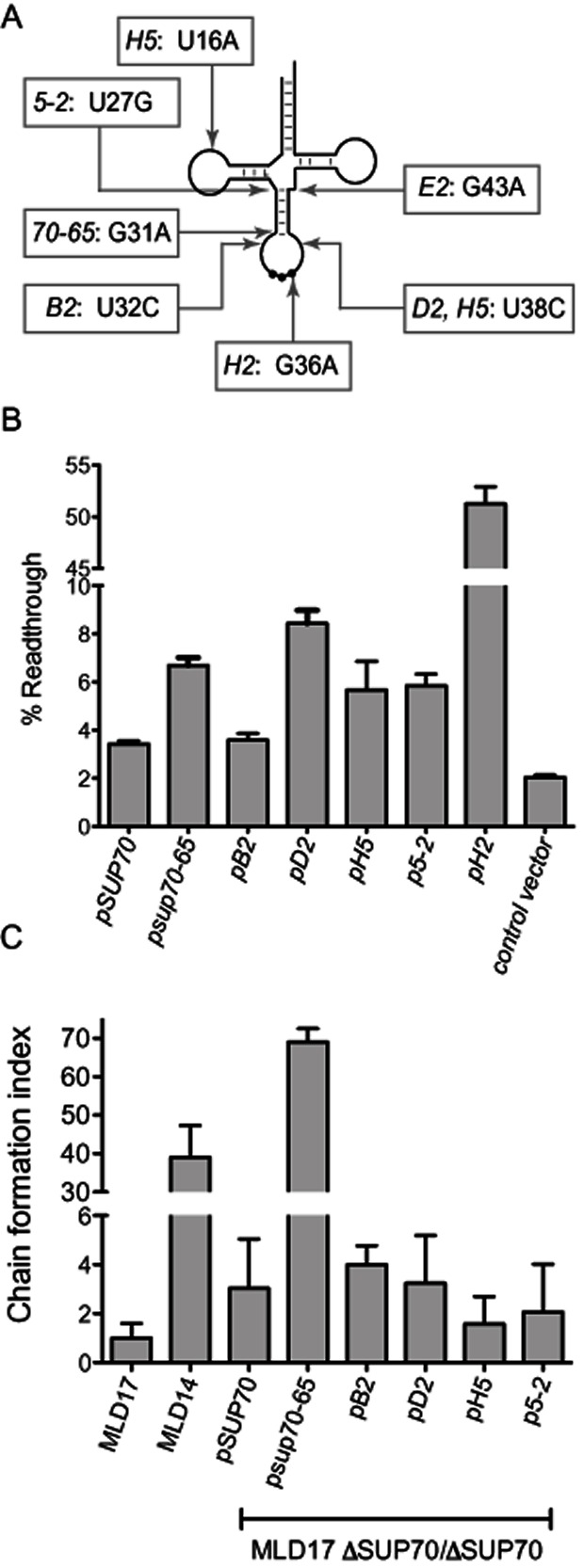
Novel *SUP70* amber suppressor mutations identify anticodon stem and loop positions that regulate first codon-position wobble. A. Location of novel amber suppressor *SUP70* mutations, all of which are single mutants with the exception of H5. All suppressed the *trp1-1* allele, causing tryptophan prototrophy in strain MLD17. Mutant E2 was not further analysed. B. Quantification of nonsense codon readthrough using a dicistronic vector system. Dominant *SUP70* alleles with amber suppressor activity were transformed into a wild-type strain (BY4743) along with a vector carrying a dicistronic stop codon readthrough assay system to quantify stop codon readthrough in three independent cultures. Error bars represent ± 1 standard deviation. C. The ability of the novel nonsense suppressor *SUP70* alleles (A; excluding mutant H2 which could not support viability), and that of *SUP70* and *sup70-65* alleles to trigger pseudohyphal chain formation was assessed by shuffling the plasmid-borne alleles into strain MLD17-Δ*SUP70* (Δ*SUP70/*Δ*SUP70*) and measuring their chain formation index (CFI) in triplicate independent transformants, grown in SD medium. Error bars represent ± 1 standard deviation.

Wild-type tRNA_CUG_ can only very inefficiently decode UAG codons via first base wobble, because *SUP70* amber (UAG) suppressor activity is detectable only when overexpressed on a multi-copy plasmid (Pure *et al*., 1985). However, the *sup70-65* mutation is a single-copy UAG suppressor (Murray *et al*., 1998) indicating the mutant tRNA has an enhanced ability to wobble-decode U in the first codon position. The *sup70-65* mutation creates an A–C nucleotide mismatch at the base of the tRNA_CUG_ anticodon stem (base pair nucleotides 31–39; Fig. 3). This presumably distorts the anticodon stem and increases the propensity to decode UAG via first base wobble. In order to identify further *sup70* mutants capable of inducing pseudohyphal growth, we wanted to identify additional *sup70* nonsense suppressor alleles. In this way, we sought to test the hypothesis that structural modifications that alter *SUP70* tRNA^Gln^_CUG_ presentation of the anticodon might generally cause a tRNA defect that triggers deregulated pseudohyphal growth.

A library of plasmid-borne tRNA^Gln^_CUG_ mutants was therefore generated via PCR. This library was transformed into wild-type strain MLD17 that carries the *trp1-1* amber mutation (UAG) in homozygous form and is thus tryptophan (Trp) auxotrophic. Trp prototrophic transformants identified *sup70* nonsense suppressor alleles. Sequencing of these alleles revealed that almost all the amber suppressor mutants defined nucleotide substitutions in the anticodon stem and loop (Fig. 3A). Stem mutations disrupt base pairing and would be predicted to disrupt structure and orientation of the anticodon loop. Reassuringly, the *sup70-65* mutation was re-isolated from the screen, as was a mutation creating a UAG-cognate anticodon (H2; Fig. 3A).

The nonsense suppressor phenotype of these novel alleles was quantified using a dicistronic readthrough assay in strain BY4743 (*SUP70/SUP70;* Fig. 3B). A UAG stop codon, placed in a poor nucleotide context for termination to increase the sensitivity of readthrough detection, separated the *lacZ* and firefly luciferase open reading frames in the readthrough assay vector. A baseline readthrough efficiency of 2% was measured in untransformed BY4743 (data not shown), due to the leakiness of the UAG codon used, and the natural propensity of *SUP70* tRNA to decode UAG via first base wobble (Weiss *et al*., 1987). An additional copy of wild-type *SUP70* increased readthrough levels to more than 3% (Fig. 3B). Excluding H2, the other mutants exhibited varying UAG suppressor efficiencies of between 4% and 9% confirming their UAG suppressor phenotypes. As expected, readthrough levels of mutant H2 were high, as it has a mutated anticodon 5′-CUA-3′ able to directly recognize the UAG stop codon. The *sup70-33* mutation, although not a suppressor screen isolate, was also tested for readthrough activity since it also exhibits a pseudohyphal growth phenotype (Murray *et al*., 1998). As expected this allele was no more effective a suppressor than the wild-type *SUP70* allele (data not shown).

Having isolated a family of novel *sup70* amber suppressor alleles, their ability to trigger un-regulated pseudohyphal differentiation was then tested by shuffling the plasmid-borne alleles into the MLD17 homozygous Δ*SUP70* knockout strain. However, with the exception of the control *sup70-65* allele, which exhibited a high chain formation index, none of the newly identified mutants induced pseudohyphal growth (Fig. 3C). We did note however that when a homozygous *SUP70* deletant is supported by a single plasmid-borne copy of the *SUP70* gene, the chain formation index was increased, indicating that reducing the gene copy number of the wild-type tRNA_CUG_^Gln^ tRNA can also establish low levels of chain formation. Since nonsense suppressor *sup70* alleles must exhibit an altered presentation of the CUG anticodon in such a way as to enhance G–U wobble base pairing at the first codon position, it was concluded that altered anticodon presentation is in itself insufficient to trigger pseudohyphal growth. The *sup70-65* tRNA must therefore exhibit other defects in addition to its altered anticodon presentation.

### Site-directed *sup70* mutants identify tRNA structural rigidity and translational efficiency as important determinants of the pseudohyphal phenotype

**Fig. 4:**
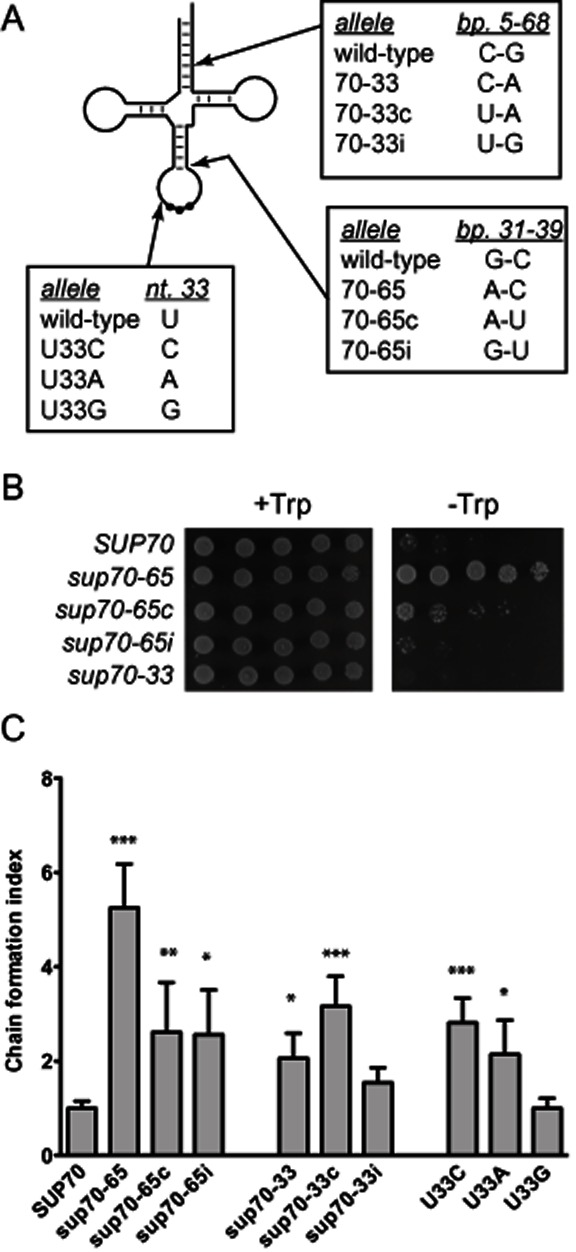
Site-directed *sup70* mutants identify tRNA structural rigidity and translational efficiency as important determinants of the pseudohyphal phenotype. A. Site-directed mutagenesis of the *SUP70* gene encoding tRNA^Gln^_CUG_ created novel mutations at the site of the *sup70-65* (base-pair 31–39) and *sup70-33* (base-pair 5–68) mutations. Mutations were also created at nucleotide 33, ordinarily a uridine residue in almost all tRNA species, and known to be important for codon–anticodon recognition and translational efficiency. B. The *sup70-65*-series site-directed mutants were assessed for their ability to suppress the *trp1-1^UAG^* nonsense allele, by transformation into MLD17-Δ*SUP70* (Δ*SUP70/*Δ*SUP70 trp1-1/trp1-1*) and assessment of growth on medium ± tryptophan. C. Plasmids were shuffled into strain MLD17-Δ*SUP70* (Δ*SUP70/*Δ*SUP70*) and their ability to induce pseudohyphal growth assessed in triplicate SD medium cultures, using the chain formation index (CFI). Error bars represent ± 1 standard deviation. Each of the bars was compared with the *SUP70* control (left hand bar); significant differences are recorded by an asterisk above the bar (*P = 0.05, **P = 0.025, ***P ≤ 0.005).

In order to characterize the impact of the *sup70-65* and *sup70-33* mutations on the function of tRNA^Gln^_CUG_, we created site-directed mutants (*sup70-65c; sup70-33c*) in which the substituted mutant nucleotide was preserved, but stem base pairing was restored, for example creating a, A_31_–U_39_ pair to create allele *sup70-65c* (Fig. 4A). Other variants were created in which a G–U wobble base pair interaction was created, to weaken stem interaction (*sup70-65i, sup70-33i*). Using these novel alleles, the role of nucleotide pair identity, and pair strength on the pseudohyphal growth phenotype could be tested.

First, we assessed the ability of the new alleles to suppress the UAG stop codon, since the *sup70-65* allele is an amber suppressor. Plasmids carrying the alleles were transformed into strain MLD17, which carries the *trp1-1^am^* UAG allele, and suppression assayed on medium lacking tryptophan (Fig. 4B). As expected, whereas the *sup70-33* allele exhibited no measurable suppressor activity, the *sup70-65* allele was an efficient suppressor. Repair of the *sup70-65* tRNA anticodon stem by nucleotide substitution to create an A_31_–U_39_ pair did not however completely eliminate suppressor ability (*sup70-65c*: Fig. 4B), while in contrast the *sup70-65i* (G_31_–U_39_) mutant exhibited no suppressor activity. Thus nucleotide identity, as well as the existence of an intact stem, appears to affect anticodon presentation and thus first base wobble.

The pseudohyphal phenotypes of the novel mutants were subsequently tested by shuffling them into in a homozygous Δ*SUP70* strain background (Fig. 4C). The results showed that recreating a position 31–39 base-pair in the *sup70-65*c and *sup70-65i* alleles significantly reduced the formation of pseudohyphae, but did not completely restore the wild-type phenotype, indicating that base pair strength and/or nucleotide identity may be important determinants of the pseudohyphal growth phenotype. Likewise in the *sup70-33* family mutants, restoration of base pairing at the 5–68 base-pair did not restore a wild-type phenotype, again indicating a role for base identity or the structural rigidity of the acceptor stem in signalling pseudohyphal responses.

Finally, we created three *SUP70* mutants at tRNA base 33, a base position that is uridine in almost all tRNAs and which when mutated markedly degrades translational efficiency (Fig. 4A) (Santos *et al*., 1996). U33 may also be a recognition determinant for the glutaminyl-tRNA synthetase (Hayase *et al*., 1992). We reasoned that if inefficient CAG codon translation was the cause of the *sup70-65* and *−33* phenotypes (perhaps in the case of *sup70-65* caused by anticodon loop distortion), then reconstituting poor decoding of CAG codons, or inefficient tRNA_CUG_^Gln^ charging, via mutation at position 33 should also generate a pseudohyphal growth phenotype. The results did indeed confirm this; although the U33G mutant did not differ from the wild-type, both U33C and U33A mutants generate significantly more pseudohyphal cell chains than the wild-type (Fig. 4C).

From these findings, we conclude that tRNA structure, as well as the identity of specific nucleotides, influences both stop codon readthrough and the regulation of pseudohyphal growth. The results also provide a preliminary indication that reductions in the translational efficiency of tRNA^Gln^_CUG_ may cause unregulated pseudohyphal growth.

### Overexpression of either tRNA^Gln^_CUG_ or tRNA^Gln^_UUG_ attenuates pseudohyphal differentiation

**Fig. 5:**
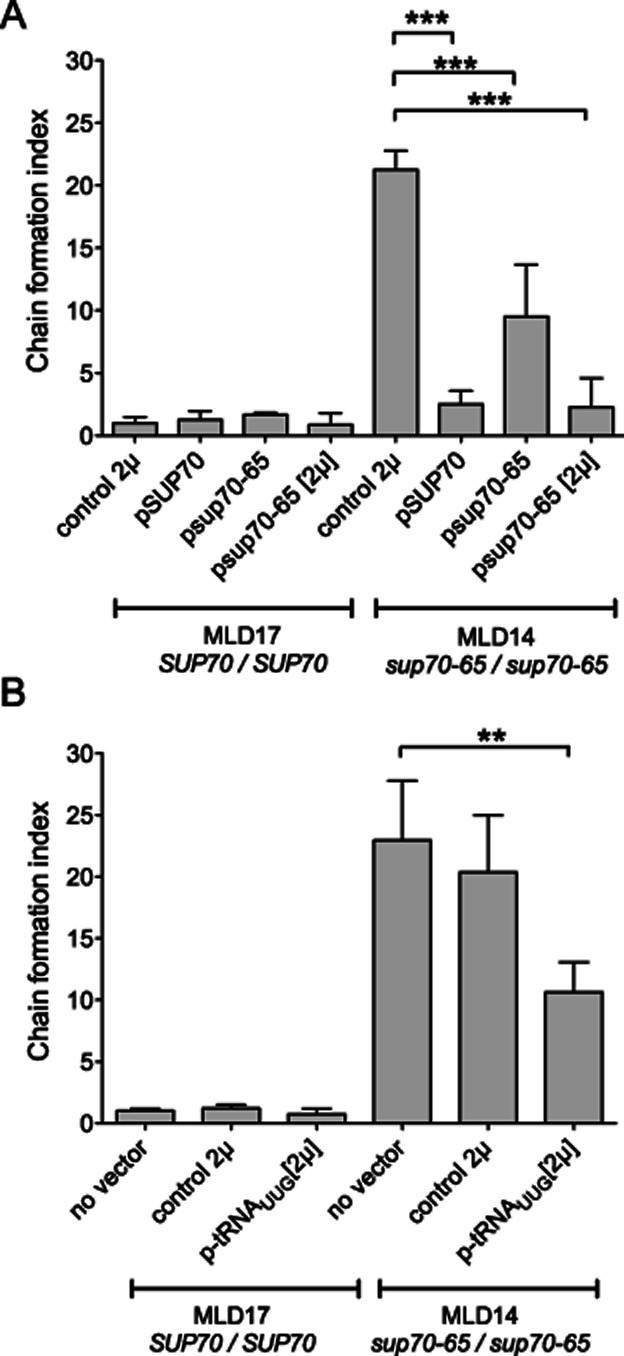
Complementation of the pseudohyphal growth defect by additional copies of tRNA^Gln^_CUG_ or tRNA^Gln^_UUG_. A. Strains MLD17 and MLD14 were transformed with either a control multi-copy vector (2μ), a single-copy vector carrying the wild-type *SUP70* gene (pSUP70), a single-copy vector carrying the *sup70-65* allele (psup70-65) or a multi-copy vector psup70-65 [2μ] carrying the *sup70-65* allele. B. Strains MLD17 (*SUP70/SUP70*) and MLD14 (*sup70-65/sup70-65*) were transformed with either a control multi-copy vector or a multi-copy vector p-tRNA_UUG_[2μ] carrying the tRNA^Gln^_UUG_ gene tQ(UUG). Pseudohyphal growth was quantified using the chain formation index, in triplicate independent transformant cultures. Error bars represent ± 1 standard deviation. Significance was calculated using a Student's *t*-test (*P = 0.05, **P = 0.025, ***P ≤ 0.005).

Since alterations in tRNA structural rigidity, anticodon loop presentation and overall translational efficiency all trigger the pseudohyphal growth phenotype, it was hypothesized that the *sup70* pseudohyphal phenotype is caused by a defect in the ability of tRNA_CUG_ to efficiently decode its cognate CAG codon. That being the case, it should be possible to repair the pseudohyphal growth phenotype by increasing the gene dosage of *sup70-65* alleles. This was found to be the case, since introducing one extra *sup70-65* gene copy on a centromeric plasmid into a *sup70-65* mutant partly repaired the phenotype, and introducing multiple extra copies using a 2μ-based vector completely repaired the phenotype (Fig. 5A).

Third base U-G wobble is thought to be restricted in eukaryotes, thus the CAA-decoding tRNA_UUG_ is a poor decoder of CAG codons. However, it is known that if overexpressed, the CAA-decoding tRNA_UUG_^Gln^ can suppress a *SUP70* (tRNA_CUG_^Gln^) gene knockout in yeast, because the mcm^5^-s^2^-modified UUG anticodon has nonetheless some weak ability to third-base wobble-decode the CAG codon (Johansson *et al*., 2008). We therefore tested whether driving improved CAG codon translation in a *sup70-65* mutant via overexpression of tRNA^Gln^_UUG_ (gene tQ(UUG)C) on a multi-copy plasmid could suppress constitutive pseudohyphal growth.

Strain MLD14 (*sup70-65/sup70-65*) and the wild-type counterpart MLD17 (*SUP70/SUP70*) were therefore transformed with the multi-copy plasmid ptRNA-UUG carrying tQ(UUG)C and were assessed for pseudohyphal growth (Fig. 5B). The results show clearly that whereas the control vector left the chain formation index unaffected, transformation of the *sup70-65* homozygous diploid with a multi-copy plasmid encoding the CAA-decoding tRNA significantly reduced pseudohyphal growth (Fig. 5B). These experiments suggested that pseudohyphal growth was being caused by the inefficient translation of CAG glutamine codons.

### tRNA_CUG_ levels in *sup70* pseudohyphal mutant strains are markedly reduced in comparison with wild-type

**Fig. 6:**
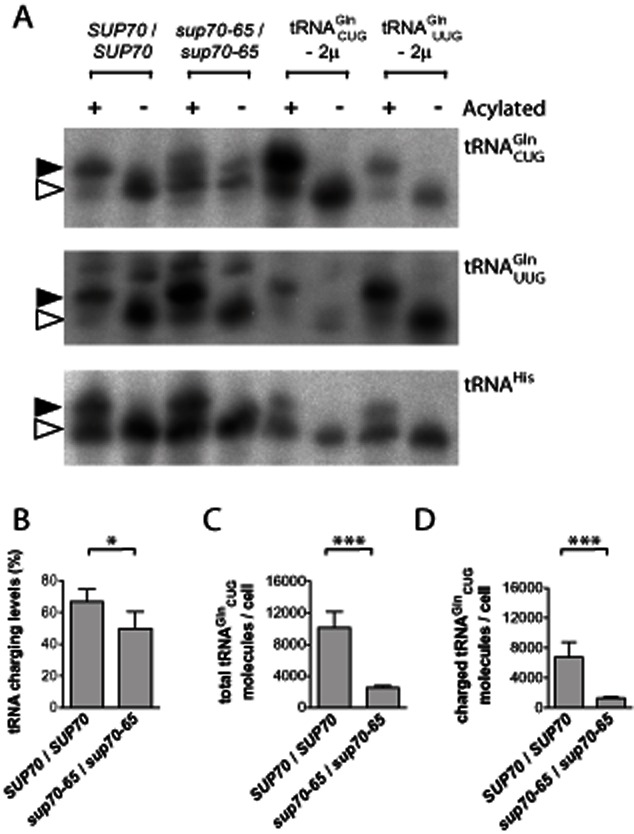
Northern blot analysis of *sup70-65* tRNA charging levels. (A) Northern blot analysis of tRNA preparations from *sup70* mutants and corresponding wild-type progenitors. A single blot was probed successively with oligonucleotides specific for tRNA^Gln^_CUG_, tRNA^Gln^_UUG_ and tRNA^His^. tRNA samples were run in both acylated and deacylated states to enable identification of charged (black arrowheads) and uncharged (open arrowheads) tRNA migration positions. tRNA extracts were prepared from strains overexpressing tRNA^Gln^_CUG_ and tRNA^Gln^_UUG_ respectively (labelled ‘2μ’), and were blotted to demonstrate the specificity of each of the probes against the two similar Gln tRNAs. To avoid over-intense probe binding, these overexpressed samples were run at 20% of the concentration of the MLD14 and MLD17 samples. One example blot is shown. B–D. Phosphoimager quantification of repeat Northern blots, employing replicate tRNA samples from independent cultures (*n* = 3), was used to identify the proportion of charged tRNA^Gln^_CUG_ in the *sup70-65* mutant and wild-type strain (B). Probing for tRNA^His^ was used to normalize tRNA loadings. Northern dot-blots loaded were with triplicate independent tRNA samples (data not shown), and a standard curve of known quantities of *in vitro* transcribed tRNA^Gln^_CUG_. These were used to quantify total quantities of tRNA^Gln^_CUG_ in mutant and wild-type strains (C). The dot-blots were re-probed for tRNA^His^ to normalize tRNA loadings. By combining the data in (B) and (C), an estimate of the total number of molecules of charged tRNA^Gln^_CUG_ in mutant and wild-type strains could be derived (D). Error bars represent ± 1 standard deviation. Significance was calculated using a Student's *t*-test (*P = 0.05, **P = 0.025, ***P ≤ 0.005).

The evidence presented so far suggested that the *sup70-65* tRNA might in some way be compromised in its ability to decode CAG codons, perhaps because its stability or charging level were affected by the mutation in the anticodon stem. We therefore used alkaline acrylamide denaturing gels, and Northern blot analysis to examine overall levels of tRNA_CUG_^Gln^ as well as its levels of glutamine charging. The more abundant isoacceptor tRNA^Gln^_UUG_ as well as the unrelated tRNA^His^ were analysed as control tRNAs, the latter also being used to normalize tRNA^Gln^_CUG_ charging levels between samples (Fig. 6).

For reliable Northern blot analysis of both yeast glutaminyl tRNAs, it was important to first verify that the oligonucleotide probes used did not cross-react, since the nucleotide sequences of tRNA^Gln^_CUG_ and tRNA^Gln^_UUG_ differ by only four nucleotides, including the anticodon difference. Accordingly, we overexpressed the genes encoding tRNA^Gln^_CUG_ and tRNA^Gln^_UUG_ in strain MLD17, and using probes specific for two of the four nucleotide differences, showed clearly that when tRNA^Gln^_CUG_ was overexpressed, no additional tRNA^Gln^_UUG_ was detected by the tRNA_UUG_ probe. The converse was also true, indicating the probes were specific under the conditions used (Fig. 6A, lanes 5–8).

Using these two probes and one for tRNA^His^, Northern blot analysis was conducted. The results showed that tRNA^His^ and tRNA_UUG_^Gln^ species existed predominantly in the charged form in both wild-type and mutant strains, and could be deacylated using alkaline conditions to produce a marked tRNA band-shift (Fig. 6A, lanes 1–4). However, detection of tRNA_CUG_^Gln^ revealed that in the *sup70-65* mutant, but not in the wild-type, probing for the mutant tRNA consistently detected a diffuse smear that was of much lower intensity than in the wild-type strain (Fig. 6A, lanes 3 and 4, top panel). Within this smear, 3–4 more faint bands could be weakly discerned, although this was blot dependent. Following deacylation, two bands were still detectable, indicating at least two separately migrating species of different mass. These results indicated that the *sup70-65* mutation had rendered the mutant tRNA^Gln^_CUG_ less abundant, and judged by the difficulty in resolving it as a single band on a denaturing gel, possibly degraded and existing in multiple forms.

Quantification of this Northern blot analysis, together with additional Northern slot blots of the mutant MLD14 (*sup70-65/sup70-65*) and MLD17 (*SUP70/SUP70*) strains (data not shown) revealed that in the mutant, charging levels are slightly reduced from 67% in the wild-type MLD17 strain, to 50% in the *sup70-65* mutant (Fig. 6B). However, importantly, total tRNA^Gln^_CUG_ levels in the mutant are reduced to 25% of that of wild-type MLD17 (Fig. 6C). Another *sup70-65* strain, LMD651, and strain LMD6533LU, carrying an allele *sup70-33* that is also known to produce uncontrolled pseudohyphal growth (Murray *et al*., 1998) were also analysed by Northern blot and again found to contain greatly reduced concentrations of tRNA_CUG_^Gln^ relative to the control tRNA_UUG_^Gln^ (Supplementary Fig. S2).

The reduced stability and charging of the mutant tRNA may be caused simply by resultant instability of the anticodon (*sup70-65*) or acceptor (*sup70-33*) stems, possibly enhanced by altered patterns of tRNA nucleoside modification. The combination of reduced overall tRNA level and reduced charging would reduce the overall level of charged tRNA available for CAG decoding in the *sup70-65* mutant to 19% of wild-type (Fig. 6D), likely to have a significant effect on CAG codon translation.

### The *sup70-65* mutant exhibits reduced expression of reporters containing tandem CAG codons

The Northern blot analysis presented in this work suggests the *sup70-65* mutant may be significantly compromised in its ability to efficiently translate CAG codons. We therefore wanted to test whether the introduction of additional tandem CAG codons at the 5′ end of the firefly luciferase ORF would reduce luciferase expression. We reasoned that slow CAG codon translation would cause extensive ribosomal queuing in the 5′ untranslated region back to the 5′ end, negatively impacting upon recruitment of ribosomal subunits by the 5′ cap structure. Less firefly luciferase would therefore be expressed.

**Fig. 7:**
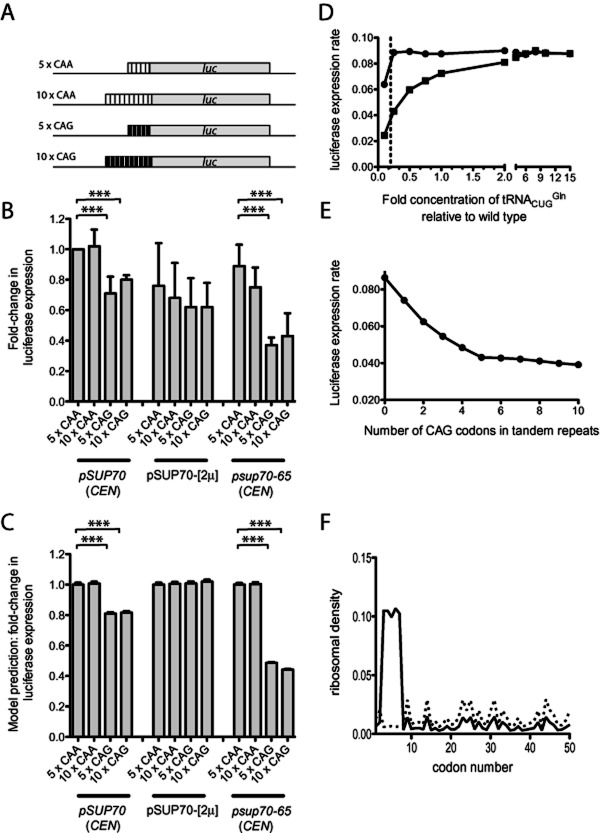
Slow translation of CAG, but not CAA, codons in a *sup70-65* tRNA mutant background. A. Plasmid-borne luciferase genes were engineered to contain either 5 or 10 tandem copies of the CAA codon at the 5′ end of the ORF (5 × CAA, 10 × CAA). Counterpart plasmids express luciferase engineered with similar CAG codon arrays (5 × CAG, 10 × CAG). B. Tandem CAA- or tandem CAG-luciferase constructs were transformed into IS700 derivative yeast strains (Δ*SUP70/*Δ*SUP70*). IS700 transformants were supported with plasmid pSUP70 (encoding tRNA_CUG_, single-copy *CEN* plasmid), p*SUP70*-[2μ] (encoding tRNA_CUG_, multi-copy plasmid) or p*sup70-65* (encoding tRNA_CUG_ [G31A], single-copy *CEN* plasmid). Luciferase enzyme levels were normalized using qRT-PCR measurement of luciferase mRNA, (*ACT1* qRT-PCR used as loading control). Normalized luciferase levels were expressed relative to the 5 × CAA construct value for the wild-type strain. Error bars represent ± 1 standard error (*n* = 5). Significance was calculated using a Student's *t*-test (*P = 0.05, **P = 0.025, ***P ≤ 0.005). C. Ribosome flux along the tandem CAA or CAG constructs was simulated in the different tRNA backgrounds, using a dynamic TASEP model of translation elongation, responsive to individual tRNA concentrations. Luciferase production rates were normalized relative to the 5 × CAA construct value for the wild-type strain. Error bars represent ± 1 standard error (*n* = 1000 simulations). D–F. In further simulations, the luciferase production rate in the *sup70-65* mutant was determined in response to different tRNA_CUG_^Gln^ concentrations in the 5 × CAA (circles) and 5 × CAG variants (squares; D); the vertical dotted line represents the level of tRNA_CUG_^Gln^ measured experimentally in the *sup70-65* mutant background. The response of luciferase expression to different numbers of CAG codons in the tandem repeats in the *sup70-65* background was also simulated (E). The ribosome density in the first 50 codons of luciferase was then recorded during simulations of translation in the *sup70-65* mutant using either the 5 × CAA construct (dashed line) or the 5 × CAG construct (solid line).

We accordingly constructed variants of firefly luciferase, with either 5 or 10 tandem copies of CAG codons at the 5′ end of the reporter ORF, and as a control, additional luciferase variants with 5 or 10 tandem CAA codons at their 5′ end (Fig. 7A). Luciferase expression was measured in five independent cultures, along with expression levels of luciferase mRNA using qRT-PCR, the latter used to normalize luciferase activity levels. Three different Δ*SUP70* knockout strains were employed, one supported by a wild-type *SUP70* gene on a centromeric vector, one carrying a multi-copy *SUP70* plasmid, and one carrying the *sup70-65* gene on a *CEN* vector.

To inform this experimental investigation and aid the analysis of the results, we also employed a recently developed mathematical model of the translation elongation process, responsive to codon-specific translation rates caused by differing tRNA abundances (Ciandrini *et al*., 2010). Using this we simulated the translation of firefly luciferase with either 5 or 10 tandem CAA or CAG codons introduced at the 5′ end of the ORF, and using a median translation initiation rate typical for yeast (0.1 s^−1^), derived from a genome-wide application of the model (L. Ciandrini, I. Stansfield, M.C. Romano, unpubl. work). The modelling of translation in the strain carrying the *sup70-65* gene was achieved by reducing the simulated levels of tRNA_CUG_ fivefold according to the results of the Northern analysis (Fig. 6), and that of the multi-copy *SUP70* strain by increasing simulated tRNA_CUG_^Gln^ abundance 15-fold, in the typical range for a 2μ vector. The model predictions could then be directly compared with experimentally measured luciferase expression levels.

The results show clearly that tandem CAG codons at the 5′ end of luciferase reduce its expression in a wild-type cell significantly, by approximately 20%, as expected given the ninefold lower abundance of the CAG-decoding tRNA relative to the CAA-decoder tRNA_UUG_ (Fig. 7B). This was true whether 5 or 10 tandem CAG codons were incorporated. This result was however also predicted by the model simulation of translation (Fig. 7C), indicating that even 5 CAG codons at the 5′ end of an ORF are sufficient to cause significant queuing, which impacts upon ribosomal recruitment. The modelling also supported the experimental observation that queuing could not be further enhanced by the introduction of additional CAG codons.

This reduced expression should, we predicted, be reparable by overexpressing the *SUP70* gene, boosting the copy number of tRNA_CUG_^Gln^ and reducing or eliminating ribosomal queuing spanning the 5′ end of ORF and 5′UTR regions. Indeed this was the case; although luciferase expression overall was slightly reduced in this strain relative to the wild-type, nevertheless the 10 × CAG-luciferase expression levels were almost restored to those of 10 × CAA-luciferase when the yeast was transformed with a multi-copy *SUP70* plasmid. Again, this experimental result was confirmed in the model simulation (Fig. 7B and C). Importantly, although expression of the 5 × CAA and 10 × CAA controls in a *sup70-65* background was little different from that in the wild-type strain, multiple tandem CAG codons in the mutant caused an even more marked inhibitory effect than was apparent in a wild-type cell, reducing luciferase expression to 40% of the control, corresponding CAA constructs (Fig. 7B). Simulating the reduced abundance of *sup70-65* tRNA using the mathematical model also predicted a similar reduction in luciferase expression, to 40% of control (Fig. 7C).

Several observations warranted further explanation. First, we noted that the correspondence between the amount of active tRNA_CUG_^Gln^ available for CAG decoding in the *sup70-65* mutant (20% of wild-type; Fig. 6), and the consequential effect on expression of the (CAG)n-*luc* reporters (approximately 40% of the corresponding CAA construct; Fig. 7B) was non-proportionate. We argued the reason for this must lie in the dynamics of the ribosomal queues forming at CAG codons. What causes inhibition of translation is not the number of inhibitory codons *per se*, but rather whether a queue of ribosomes forms, and stretches back to the 5′ mRNA cap to inhibit the joining of new ribosomes. The extent of the queue that forms is a product of a delicate balance between the rate of translation initiation, defining how fast ribosomes are joining the queue, and the rate with which ribosomes bypass the run of slow codons and leave the queue. Added to this, how fast ribosomes translate through a CAG array is difficult to predict simply on the basis of tRNA_CUG_ concentration; while a ribosome *x* at a CAG codon is stalled by another downstream ribosome *y* also at a CAG codon and waiting to encounter a tRNA_CUG_^Gln^ the more 5′ ribosome *x* may in fact encounter the correct tRNA first and thus become ‘unblocked’. Inhibition of translation by tandem codon arrays is a complex function of array size, tRNA concentration and distance from 5′ cap to array.

Using the computer model, we simulated the effect of a range of tRNA_CUG_ concentrations on luciferase production from the 5 × CAA and 5 × CAG constructs, and first confirmed that as measured experimentally, 5 × CAA luciferase expression was essentially unresponsive to changes in tRNA_CUG_ abundance. 5 × CAA luciferase expression was in fact only affected when tRNA_CUG_ was reduced to extremely low levels, significantly below the 0.2-fold wild-type level of tRNA_CUG_^Gln^ (indicated by the dotted line) experimentally measured in the *sup70-65*. The reason why very low levels of tRNA_CUG_^Gln^ can affect expression of 5 × CAA is because of the presence of the seven internal CAG codons scattered across the native *luc* reading frame.

Importantly, we observed that as expected, 5 × CAG construct luciferase expression was extremely responsive to changes in tRNA_CUG_^Gln^ levels. However, because of the complex dynamics of ribosome transit through runs of tandem codon repeats, the relationship between tRNA_CUG_ concentration and luciferase 5 × CAG-luciferase expression was indeed non-linear (Fig. 7D).

Second, we noted that 10 × CAG codons were no more inhibitory to luciferase mRNA translation than 5 × CAG codons (Fig. 7B and C). We reasoned that the effect of additional CAG codons must eventually saturate, since some minimum number would be sufficient to establish a queue stretching back to the 5′ mRNA cap, thus inhibiting the translational rate. To confirm this, using *sup70-65* levels of tRNA_CUG_^Gln^, we simulated the effect of gradually adding CAG codons to the 5′ end of the luc open reading frame. The results confirm that the inhibitory effect of incrementing the number of CAG codons is additive up to *n* = 5, but there is a point of inflection at *n* = 5, beyond which additional CAG codons effectively do not increase inhibition (Fig. 7E), explaining why there was little difference between the inhibitory effect of the 5 × CAG and 10 × CAG constructs in the experimental analysis (Fig. 7B). The queuing effect was confirmed by plotting the ribosomal density across the first 50 codons of the 5 × CAA and 5 × CAG constructs, showing that the tandem CAG variant had a far higher ribosomal density across the codon repeats (codons 2–14) than did the tandem CAA array (Fig. 7F).

Thus in summary CAG decoding efficiency is significantly compromised in the *sup70-65* mutant background, exactly as predicted by the results of the tRNA Northern blot and mathematical model analysis, leading to highly significant reductions in the expression of genes containing tandem 5′ CAG codons.

## Discussion

Transfer RNA molecules play a key role in delivering amino acids from the cytoplasmic pool to the polysomes engaged in mRNA translation. Such a central process has obvious potential to control the flux of ribosomes on the mRNA, and thus, protein expression. However, with some notable exceptions such as the *Streptomyces bldA* tRNA mutants, the study of how gene expression can be regulated via tRNA effects on translation elongation has been somewhat neglected. To address this, in this work we have undertaken a detailed characterization of the mechanism behind the observation that certain mutants of the *SUP70* gene, encoding yeast tRNA_CUG_^Gln^, can promote growth of the organism in pseudohyphal-like chains of cells (Murray *et al*., 1998). The original report identifying this novel link between the translational apparatus and a developmental process regulating cell shape successfully excluded some possible molecular mechanisms – for instance, showing tRNA_CUG_ effects are independent of the *STE* signalling pathway – but did not identify the molecular mechanism, speculating that the tRNA_CUG_ may have a non-translational signalling role (Murray *et al*., 1998). Here we assemble a body of evidence that strongly suggests the contrary; that *sup70-65* tRNA_CUG_ is defective in its ability to efficiently translate the CAG codon, and that the *sup70-65* mutation represents an important example of how mutations in single-copy tRNAs can regulate specific phenotypes in eukaryote cells.

Early on in this investigation we determined that the pseudohyphal growth phenotype triggered by the *sup70-65* tRNA mutation is atypical. The MLD14 homozygous *sup70-65* diploid used in this work formed pseudohyphae constitutively in nitrogen-replete, rich liquid medium, but not on solid medium. This is quite distinct from the phenotype exhibited by the Σ1278b strain background, which forms foraging projections of cell chains that radiate outwards from colonies on nitrogen-limited solid SLAD medium (Gimeno *et al*., 1992). Using live cell microscopy monitoring, our work showed that *sup70-65* mutants form chains of cells via bipolar budding, and not in a unipolar manner as described for Σ1278b pseudohyphae. Although further characterization of the *sup70-65* phenotype was beyond the scope of this study, it appears as if the chains of cells formed by the tRNA mutant may be more typical of the fusel alcohol-triggered pseudohyphae signalled via the Swe1 morphogenesis checkpoint, since fusel alcohols also produce pseudohyphal growth on complex liquid media (Dickinson, 1996; 2008; Martinez-Anaya *et al*., 2003). This would explain why there was no evidence for *STE* pathway involvement in *sup70-65* chain formation (Murray *et al*., 1998).

One key finding of this work was the discovery that relative to a wild-type cell, tRNA_CUG_ in the *sup70-65* mutant is present at much reduced abundance (fivefold less), probably arising through instability of the mutant form of the tRNA (Fig. 6). Moreover, the pseudohyphal growth phenotype can be complemented by overexpression of tRNA_UUG_ (Fig. 5), which is known to be able to inefficiently decode the CAG codon, albeit inefficiently (Johansson *et al*., 2008). This hints strongly that CAG codons are being decoded slowly in the *sup70-65* mutant, simply because this already rare tRNA_CUG_ is even further depleted. Further support for this hypothesis is provided by our observation that the pseudohyphal growth phenotype is even more marked (exhibiting larger chain formation indices) in a homozygous *SUP70* deletant diploid carrying a single plasmid borne gene copy of *sup70-65*, clear evidence of haplo-insufficiency (Fig. 3C).

In order to test the above conclusion more directly, we used reporter genes engineered to contain additional CAA or CAG codons; consistent with the hypothesis of slowed CAG translation, we show first that introduction of multiple, tandem CAG codons at the 5′ end of a firefly luciferase reporter ORF causes significant, 20%, decreases in expression level of the reporter, even in a wild-type cell. This is simply because the CAG-decoding tRNA is ninefold less abundant than CAA-decoding tRNA, inducing ribosomal queues at the 5′ end of the mRNA. Other researchers have also demonstrated that the introduction of multiple tandem CAG codons into the 5′ end of the luciferase ORF resulted in reduced translational expression in wild-type *S. cerevisiae* (Letzring *et al*., 2010). When the experiment was repeated in a *sup70-65* mutant, expression of the CAG-engineered reporter was further reduced by 60% relative to the level achieved in a CAA-containing control construct (Fig. 7). A stochastic model of ribosomal dynamics with a single-codon resolution (Ciandrini *et al*., 2010) (L. Ciandrini, I. Stansfield and M.C. Romano, unpubl. work) has been used to simulate the translation of the engineered constructs. The results of the *in silico* translation are strongly consistent with the experimental results (Fig. 7C), and support the hypothesis that slow CAG decoding is the main cause of reduced expression of the codon-engineered luciferase genes in the *sup70-65* mutant.

Curiously, in the original publication describing the properties of the *sup70-65* tRNA, Murray *et al*. engineered a *lacZ* reporter gene to contain extra tandem CAG copies, and reported no difference in β-galactosidase expression between *sup70-65* mutant and wild-type yeast (Murray *et al*., 1998). We are unable to explain this difference between their results and ours, but one possibility might lie in the other group's choice of *lacZ* as a reporter. *lacZ,* a significantly longer ORF of over 1000 codons, already contains 43 CAG codons, representing a significant CAG queue threat, and this might have masked the compromised CAG codon translation we report here. In contrast, firefly luciferase only contains seven CAG codons and so is a more sensitive reporter with which to investigate CAG codon translation. In addition, these researchers showed that a hybrid tRNA comprising the backbone of the tRNA_UUG_, engineered to have a CUG anticodon, could suppress the pseudohyphal growth phenotype, and concluded that the CUG anticodon was a critical component for the (translation-independent) signal to the N-starvation pathway (Murray *et al*., 1998). This result is however entirely explicable by our data, because creation of such a hybrid tRNA will of course restore efficient CAG translation and thus complement the pseudohyphal growth phenotype.

Murray *et al*. second argue that the *sup70-65* tRNA is unlikely to signal to the N-starvation pathway via compromised CAG translation, since the mutant tRNA is an efficient suppressor of the *trp1-1* allele and must therefore be translationally competent (Murray *et al*., 1998). We too confirm that the *sup70-65* tRNA_CUG_ is an amber suppressor, despite its reduced abundance (Fig. 3), and we also agree that CAG translation must function at some level in the *sup70-65* mutant, simply because *SUP70* is an essential gene. However we argue that because nonsense suppression is a dominant phenotype, commitment to colony growth by a *trp1-1* mutant yeast could result from very efficient suppression by a very small population of tRNAs. Thus the CAG translational inefficiency we demonstrate in this study is not necessarily incompatible with nonsense suppressor ability. Indeed, a related study of *E. coli* tRNA^Gln^ mutants found some with 3 × 10^5^-fold reduced specificity constant for the *E. coli* glutaminyl tRNA synthetase. They were thus poorly charged, but were nevertheless efficient amber suppressors (Jahn *et al*., 1991).

Since *sup70-65* tRNA is both a trigger for pseudohyphal growth and an amber codon suppressor, we generated several novel nonsense suppressor tRNA^Gln^_CUG_ alleles in an attempt to isolate novel pseudohyphal growth mutants. All but one of the mutations were located directly in either the anticodon loop or the anticodon stem, identifying those tRNA nucleotides that regulate first base wobble (Fig. 4A). These results confirm a much earlier study describing an *E. coli* tRNA^Trp^ mutated to recognize a Gln CAG codon. In that paper, amber codon suppression via first base wobble could be enhanced by a range of anticodon stem mutations, many of which destabilized the host tRNA (Schultz and Yarus, 1994). The Schultz and Yarus study, and the results presented in this article, suggest that tRNA anticodons have been selected to minimize first base wobble. In our work, the screen also resulted in the independent re-isolation of the *sup70-65* mutant, confirming its previously documented nonsense suppression phenotype (Murray *et al*., 1998), as well as an anticodon mutant (H2) directly cognate for the UAG stop codon. However in a Δ*SUP70* background, none of the novel mutants triggered pseudohyphal growth. It seems as if generally distorting the anticodon stem or loop, while necessary to induce nonsense suppression, is not in itself sufficient to trigger pseudohyphal growth. *sup70-65* is therefore unusual in exhibiting a combined phenotype, its mutation altering anticodon presentation and thus first base wobble. In addition this mutation also more generally reduces tRNA_CUG_ abundance, probably through loss of tRNA stability as indicated by the Northern blot analysis (Fig. 6).

In this study, we have provided strong evidence to suggest that the pseudohyphal phenotype triggered by *sup70-65* is caused directly by a translational defect. We show first that *sup70-65* tRNA is unstable and poorly charged. Knowing that the *sup70-65* pseudohyphal growth phenotype is genetically recessive and therefore caused by loss of function, there is a clear link drawn between that phenotype and at the molecular level, a loss of the known function of tRNA in translation that we show here. Using the reporter CAG-engineered luciferase, we show that the unstable, defective tRNA is unable to translate CAG codons as efficiently as wild-type. Finally, we show that the pseudohyphal growth phenotype can be partly complemented by overexpression of tRNA_UUG_^Gln^, known to weakly decode CAG codons (Johansson *et al*., 2008). Taken together, the evidence invites a very obvious conclusion; that reduced tRNA_CUG_ abundance causes slower than normal translation of CAG codons, which in some way, signals pseudohyphal growth. The exact mechanism is however unclear; in unpublished work we have showed, for example, that *sup70-65* mutants do not exhibit an altered unfolded protein response, known to play a role in pseudohyphal growth signals (Schroder *et al*., 2000); Murray *et al*. exclude *STE* pathway signalling in their earlier work (Murray *et al*., 1998), and defects in the nitrogen catabolite response in *sup70-65* mutants were also excluded (Beeser and Cooper, 1999). We speculate instead that the *sup70-65* mutation alters the efficiency of translation of the mRNA encoding a negative regulator of pseudohyphal growth, which we predict would contain key CAG codons towards the 5′ end of its open reading frame. Slower than normal translation of these codons would generate ribosomal queues on this and other mRNAs in the mutant tRNA background, compromising the ability of that mRNA to sequester ribosomes and thus reducing the translational expression of the putative negative regulator. In addition to *Streptomyces bldA* mutants, there are other precedents for similar mechanisms of tRNA regulation of gene expression. In yeast, the Elp complex is a conserved protein assembly responsible for U34 wobble position tRNA modifications such as mcm^5^ (Huang *et al*., 2005). *Elp* mutants show defects in the DNA damage response and telomeric silencing, but these defects can be complemented by overexpressing the tRNA targets of the Elp modifications, indicating translational regulation of telomeric silencing via the degree of tRNA modification (Chen *et al*., 2011). In fission yeast, the Elp complex activity appears to control the cell cycle through translational control of the protein kinase cdr2 expression, via lysine codon usage within the *cdr2* ORF (Bauer *et al*., 2012). Thus specific alterations in the tRNA milieu, although exerting global effects on the translation apparatus, can nevertheless have defined phenotypic consequences for the expression of particular groups of genes, dependent upon codon content. Further work is ongoing in our laboratory to identify *S. cerevisiae* CAG-rich genes whose expression is *SUP70*-regulated.

## Experimental procedures

### *S. cerevisiae* strains and growth conditions

*Saccharomyces cerevisiae* strains MLD17 (*MATa/MATα trp1-1/trp1-1 ura3-52/ura3-52 his3-11/his3-11 ade1/ade1*), MLD15 (*MATa/MATα sup70-65/SUP70 trp1-1/trp1-1 ura3-52/ura3-52 leu2-3112/LEU2 his3-11/his3-11 ade1/ADE1*), MLD14 (*MATa/MATα sup70-65/sup70-65 trp1-1/trp1-1 ura3-52/ura3-52 leu2-3112/LEU2 his3-11/his3-11 ade1/ADE1*), LMDWU (*MAT*a/*MAT*α *SUP70*/*SUP70 ura3-52*/*ura3-52 leu2-3112*/*LEU2 ade1-1*/*ADE1*), LMD651U (*MAT*a/*MAT*α *sup70-65*/*sup70-65 ura3-52*/*ura3-52 leu2-3112*/*LEU2 ade1-1*/*ADE1*) and LMD6533LU (*MAT*a*/MAT*α *sup70-33/sup70-33 leu2-3112/ leu2-3112 ura3-52/ ura3-52*) were used to investigate the causes of the pseudohyphal growth phenotype. These strains were provided by Dr L. Murray and Prof. R.A. Singer (Dalhousie University, Halifax, Canada). Strain BY4743 (*MATa/MATα his3*Δ*1/his3*Δ*1 leu2*Δ*0/leu2*Δ*0 LYS2/lys2*Δ*0 met15*Δ*0/MET15 ura3*Δ*0/ura3*Δ*0*) was employed as a wild-type S288C-derivative strain.

A *SUP70* knockout diploid strain MLD17 ΔSUP70 (*MATa/MATα sup70::kanMX/sup70::natMX ade1/ade1 his3-11/his3-11 trp1-1/trp1-1 ura3-52/ura3-52* [pAK01]) supported by a plasmid-borne copy of *SUP70* was generated as follows. Using primers sup70-S1 and sup70-S2 (Table S1), heterozygous knockouts of the *SUP70* gene were created in BY4743 with either the kanMX (Wach *et al*., 1994) or natMX (Goldstein and McCusker, 1999) cassettes using the short-flanking homology method (generating strains BY4743 *sup70::kanMX* and BY4743 *sup70::natMX*). Using primers preS1 (situated in the upstream *URA10* gene) and postS2 (hybridizing within the downstream *SCS7* gene; Table S1), a kanMX cassette with *SUP70-*homologous long flanking regions was amplified from strains BY4743 *sup70::kanMX* genomic DNA and transformed into strain MLD17. Plasmid pAK01 (*URA3*, *CEN*) carrying the *SUP70* allele was then transformed into the MLD17 *sup70* heterozygous disruptant. Finally, primers preS1 and postS2 were used to amplify a natMX disruption cassette with *SUP70* long flanking regions using BY4743 *sup70::natMX* DNA as a template; this was transformed into the MLD17 heterozygous *sup70* deletant to generate strain MLD17 ΔSUP70. Antibiotic resistance, resistance to 5-fluoroorotic acid and diagnostic PCR were all used to verify successful deletion of both *SUP70* gene copies.

A second *SUP70* homozygous knockout strain supported by a plasmid-borne copy of *SUP70* was generated using strain BY4743. One allele of *SUP70* was deleted using a *SUP70*-kanMX knockout cassette as described above and support plasmid pAK01 transformed into the strain. The diploid strain was then sporulated. Two spores were selected that had geneticin-resistance, and 5-fluoroorotic acid-sensitivity (indicating dependence on pAK01 [*URA3 SUP70*]). These were mated to create IS700 (*MATa/MATα sup70::kanMX/sup70::kanMX his3*Δ*1/his3*Δ*1 leu2*Δ*0/leu2*Δ*0 lys2*Δ*0/lys2*Δ*0 met15*Δ*0/met15*Δ*0 ura3*Δ*0/ura3*Δ*0* [pAK01]).

Strains were grown at 30°C on either solid or liquid form of either nutrient-rich YPD medium (2% peptone, 1% yeast extract, 2% glucose), appropriate synthetic-defined (SD) selective dropout media (0.67% yeast nitrogen base, 2% glucose) or nitrogen-poor selective SLAD media (0.19% yeast nitrogen base without amino acids and ammonium sulphate, 2% glucose, 50 μM ammonium sulphate). Where required, 5-fluoroorotic acid, geneticin and nourseothricin were used at final concentrations of 200 μg ml^−1^, 100 μg ml^−1^ and 0.5 mg ml^−1^ respectively.

### Pseudohyphal growth assessment

Pseudohyphal growth in liquid medium was assessed as follows. From an initial overnight culture, a second 5 ml culture was grown for 16 h until an OD_600_ of 0.6 was reached. At least 200 cells were counted using a haemocytometer. Total cell counts, as well as chain length counts were recorded. In order to produce an index of chain forming ability (CFI), cell counts were binned according to their length of chain. Bin counts for chains greater than three cells were used to calculate a cumulative total that described what proportion of the population had chain length ≥ *L*.

*p*_L_ *=* total percentage of the population contained within chain length larger than or equal to *L*.

A cumulative sum *S*_C_ was then calculated as follows:


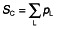


The greater a proportion of cell population that exists in long chains, the larger this ‘sum of cumulative counts’ will be. Essentially the value of *S*_C_ represents the integrated area under the cumulative sum curve. An exemplar plot of the cumulative sum showing these areas is shown in Fig. S1.

The *S*_C_ value for a given mutant was then expressed as a proportion of the corresponding wild-type *S*_C_ to produce a chain formation index, or CFI, allowing quantitative comparison of the chain formation capability.


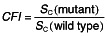


### Plasmids

Primers used in plasmid construction are itemized in Table S1. Plasmids pSUP70 (*HIS3, CEN*) carrying *SUP70,* psup70-33 (*HIS3, CEN*) carrying *sup70-33* and psup70-65 (*HIS3, CEN*) carrying *sup70-65* were created by PCR-amplifying the relevant *SUP70* allele using primers sup70-S3 and sup70-S4 (Table S1), situated approximately 190 nt 5′ and 160 nt 3′ respectively from the *SUP70* tRNA sequence, and cloning into pRS413 (Christianson *et al*., 1992) cut with NotI. Plasmids pAK01 (*URA3, CEN*) carrying the *SUP70* gene, pSUP70-2μ (*HIS3, 2μ* multi-copy) carrying *SUP70* and pSUP70-65-2μ (*HIS3, 2μ* multi-copy) carrying *sup70-65* were created by amplifying the relevant cloned *SUP70* allele with primers pRS-forward and pRS-reverse (Table S1) and using *in vivo* homologous recombination in yeast to repair NotI-gapped pRS416 or pRS423.

Plasmid pAK11 (*HIS3, CEN*) carries the *sup70* [G31A,C39T] allele (*sup70-65c*) generated using oligonucleotides atRNA-std_fw2, atRNA-std_rv2 and sup70-65c; pAK12 (*HIS3, CEN*) carries the *sup70* [C39T] allele (*sup70-65i*) generated using atRNA-std_fw2, atRNA-std_rv2 and sup70-65inv; pAK13 (*HIS3, CEN*) carries the *sup70* [C5T,G58A] allele (*sup70-33c*) generated using atRNA-7033_fw, atRNA-7033_rv and sup70-33c; pAK14 (*HIS3, CEN*) carries the *sup70* [C5T] allele (*sup70-33i*) generated using atRNA-7033_fw, atRNA-7033_rv and sup70-33inv; pAK15 (*HIS3, CEN*) carries the *sup70* [T33C] allele (*sup70-C33*) generated using atRNA-std_fw2, atRNA-std_rv2 and sup70-U33C; pAK16 (*HIS3, CEN*) carries the *sup70* [T33A] allele (*sup70-A33*) generated using atRNA-std_fw2, atRNA-std_rv2 and sup70-U33A; and pAK17 (*HIS3, CEN*) carries the *sup70* [T33G] allele (*sup70-G33*) generated using atRNA-std_fw2, atRNA-std_rv2 and sup70-U33G. For each plasmid, the oligonucleotides listed first and second were used in a 10-cycle annealing and polymerization reaction. The product of this reaction was used as PCR template in a second reaction using the second and third primers, to create a given tRNA allele ready for cloning.

Plasmid pJR5 (*LEU2, CEN*), used for quantifying stop codon readthrough, directs the expression of an in-frame translational fusion of *lacZ* and *luc* genes, separated by an in-frame UAG stop codon (pAC98-PDE2; Williams *et al*., 2004); pJR7 (*LEU2, CEN*) is identical to pJR5 but with the in-frame stop codon separating *lacZ* and *luc* genes replaced by a glutamine CAG codon.

Plasmids p5 × CAA-luc, p10 × CAA-luc, p5 × CAG-luc and p10 × CAG-luc represent yeast shuttle vectors (*TRP1 CEN*) that encode constitutively expressed firefly luciferase with tandem 5, or 10, tandem CAA, or CAG codons immediately downstream of the AUG translation initiation codon. They were created using a homologous recombination gap repair strategy in which a 5′ segment of firefly luciferase was amplified using a forward primer carrying a 5′ overhang encoding the tandem CAA or CAG codons. Using these PCR fragments, YCplac22-FL1 (*TRP1 CEN fluc*: Oliveira *et al*., 1993; Oliveira and McCarthy, 1995) cut with NdeI was gap-repaired using homologous recombination in yeast. For each PCR, the same 3′ primer was employed (lucR), partnered with either 5′ primer 5CAA-luc-F, 10CAA-luc-F, 5CAG-luc-F or 10CAG-luc-F.

### Generation of a mutant *sup70* library

The *sup70* mutant library was generated by PCR-amplifying the *SUP70* wild-type gene and approximately 400 nt of flanking vector sequence using primers pRS-forward and pRS-reverse and plasmid pSUP70 as a template (Table S1). The PCR reaction was made error-prone using non-equivalent concentrations of the dNTPs in the PCR reaction (0.144 mM dGTP, 0.144 mM dATP, 0.7 mM dTTP, 0.7 mM dCTP), combined with 0.3 mM MnCl_2_. Using both treatments ensured that a reasonable frequency of mutagenesis would be achieved within a small (72 nt) mutagenic target. The amplified, mutagenized library of *sup70* fragments was co-transformed into MLD17 (*trp1-1*) with NotI-gapped pRS413. The vector sequences flanking the tRNA gene directed homologous recombination, and thus gap repair *in vivo* of pRS413, creating a mutagenized tRNA library.

### Stop codon readthrough assays

To quantify readthrough of the UAG stop codon, yeast strains were transformed with either pJR5 or the control vector pJR7, which express β-galactosidase-luciferase fusion proteins. Dicistronic assays for stop codon readthrough were performed essentially as described (Forbes *et al*., 2007) with further modifications as detailed (Rato *et al*., 2011).

### tRNA preparation and RNA blots

tRNA preparation was performed according to standard protocols (Hill and Struhl, 1986), with some modifications (Varshney *et al*., 1991). All tRNA preparations were stored in sodium acetate buffer (pH 4.6) at −80°C until used, except those that were to be deacylated, which were ethanol precipitated, washed with 70% ethanol then resuspended in 0.2 mM Tris-acetate, pH 9.0 and incubated for 1 h at 37°C. Charged and uncharged tRNAs were resolved electrophoretically on 40 cm denaturing 10% acrylamide gel [1 M sodium acetate (pH 4.8), 10% of 40% acrylamide/bis solution (19:1), 8 M urea]. Using semi-dry blotting, tRNAs within a 20 cm lower part of the gel was then transferred onto Amersham Hybond-N membrane at 8 V, 400 mA for 1 h, and fixed to the membrane using ultra-violet irradiation (120 mJ cm^−2^). For quantification of total tRNA, 100 pmol of tRNA preparation was slot-blotted onto Amersham Hybond-N membrane using a hybridization manifold and then immediately UV-cross-linked as above.

The probes to the respective tRNAs were end-labelled using polynucleotide kinase and standard methods (Sambrook and Russell, 2001), probes purified using G-25 spin columns, and hybridized to the blot membrane using standard conditions at 42°C (Sambrook and Russell, 2001). tRNA_CUG_ was detected using a 1:1 mix of *SUP70* probe (5′-ttg ttc gga tca gaa cc-3′) and *sup70-65* probe (5′-ttg ttc gga tca gaa tc-3′). tRNA^Gln^_UUG_ was detected using the probe 5′-ttg tcc gga tca aaa cc-3′, and tRNA^His^ detected using the probe 5′-ttt cat cgg cca caa cg-3′. Washing was carried out using sequential 15 min. treatments with wash solution I (5 × SSC, 0.1% SDS), II (1 × SSC, 0.5% SDS) and III (0.1 × SSC, 1% SDS). The blot was exposed for 24–72 h to a phosphoimager screen and hybridization quantified using a Fuji FLA-3000 phosphoimager and Aida/2D v 2.0 densitometry software.

### Luciferase assays and qRT-PCR quantification of luciferase mRNA

Luciferase assays were performed on lysates of five independent yeast cell cultures grown on SD medium until an optical cell density (600 nm) of 0.8 had been reached. Assays were performed using the Bright-Glo luciferase assay kit (Promega). qRT-PCR was carried out on five independent mRNA samples (Rneasy, Qiagen) following cDNA synthesis using Quantitect reverse transcriptase (Qiagen). Then, using SYBR Green methodology in a PCR reaction (LightCycler 480 SYBR Green I Master, Roche) using a Roche LightCycler 480 RT-PCR machine, the following were performed; using primers FLuc-f and FLuc-r (Table S1), amplification of a fragment of luciferase; in a parallel reaction, using primers ACT1-f and ACT1-r (Table S1), amplification of a fragment of the *ACT1* cDNA as an internal loading control.

### Mathematical modelling of translation

An extended Totally Asymmetric Simple Exclusion Process (TASEP) model of translation that had been previously developed (Ciandrini *et al*., 2010) was used to simulate translation on any given transcript. The stochastic model describes the kinetics of ribosomes on mRNAs, mimicking their bio- and mechanochemical cycle with a two-state dynamics. Yeast codon translation rates were estimated from data on tRNA abundances, assumed to be proportional to their gene copy numbers (Percudani *et al*., 1997) and adjusted to consider further corrections such as the wobble base pairing (L. Ciandrini, I. Stansfield and M.C. Romano, unpubl. work).

For each simulation, 1000 iterations were run, and the mean and standard error values for ribosomal density and translational efficiency (ribosomal ‘current’) were recorded. The model is freely available from the authors.

## References

[b1] Bauer F, Matsuyama A, Candiracci J, Dieu M, Scheliga J, Wolf DA (2012). Translational control of cell division by elongator. Cell Rep.

[b2] Beeser AE, Cooper TG (1999). Control of nitrogen catabolite repression is not affected by the tRNAGln-CUU mutation, which results in constitutive pseudohyphal growth of *Saccharomyces cerevisiae*. J Bacteriol.

[b3] Buchan JR, Stansfield I (2007). Halting a cellular production line: responses to ribosomal pausing during translation. Biol Cell.

[b4] Chen C, Huang B, Eliasson M, Ryden P, Bystrom AS (2011). Elongator complex influences telomeric gene silencing and DNA damage response by its role in wobble uridine tRNA modification. PLoS Genet.

[b5] Christianson TW, Sikorski RS, Dante M, Shero JH, Hieter P (1992). Multifunctional yeast high-copy-number shuttle vectors. Gene.

[b6] Ciandrini L, Stansfield I, Romano MC (2010). Role of the particle's stepping cycle in an asymmetric exclusion process: A model of mRNA translation. Phys Rev E Stat Nonlin Soft Matter Phys.

[b8] Dickinson JR (1996). ‘Fusel’ alcohols induce hyphal-like extensions and pseudohyphal formation in yeasts. Microbiology.

[b7] Dickinson JR (2008). Filament formation in *Saccharomyces cerevisiae* – a review. Folia Microbiol (Praha).

[b9] Doma M, Parker R (2006). Endonucleolytic cleavage of eukaryotic mRNAs with stalls in translation elongation. Nature.

[b10] Dong H, Nilsson L, Kurland CG (1996). Co-variation of tRNA abundance and codon usage in *Escherichia coli* at different growth rates. J Mol Biol.

[b11] Forbes EM, Nieduszynska SR, Brunton FK, Gibson J, Glover LA, Stansfield I (2007). Control of gag-pol gene expression in the *Candida albicans* retrotransposon Tca2. BMC Mol Biol.

[b12] Gimeno CJ, Ljungdahl PO, Styles CA, Fink GR (1992). Unipolar cell divisions in the yeast *S. cerevisiae* lead to filamentous growth: regulation by starvation and RAS. Cell.

[b13] Goldstein AL, McCusker JH (1999). Three new dominant drug resistance cassettes for gene disruption in *Saccharomyces cerevisiae*. Yeast.

[b14] Grunberg-Manago M (1999). Messenger RNA stability and its role in control of gene expression in bacteria and phages. Annu Rev Genet.

[b15] Hayase Y, Jahn M, Rogers MJ, Sylvers LA, Koizumi M, Inoue H (1992). Recognition of bases in *Escherichia coli* tRNA(gln) by glutaminyl-tRNA synthetase: a complete identity set. EMBO J.

[b16] Hill DE, Struhl K (1986). A rapid method for determining tRNA charging levels *in vivo*: analysis of yeast mutants defective in the general control of amino acid biosynthesis. Nucleic Acids Res.

[b17] Huang B, Johansson MJ, Bystrom AS (2005). An early step in wobble uridine tRNA modification requires the elongator complex. RNA.

[b18] Ikemura T (1982). Correlation between the abundance of yeast transfer RNAs and the occurrence of the respective codons in protein genes. Differences in synonymous codon choice patterns of yeast and *Escherichia coli* with reference to the abundance of isoaccepting transfer RNAs. J Mol Biol.

[b19] Jahn M, Rogers MJ, Soll D (1991). Anticodon and acceptor stem nucleotides in tRNA(gln) are major recognition elements for *E. coli* glutaminyl-tRNA synthetase. Nature.

[b20] Johansson MJ, Esberg A, Huang B, Bjork GR, Bystrom AS (2008). Eukaryotic wobble uridine modifications promote a functionally redundant decoding system. Mol Cell Biol.

[b21] Keiler KC, Waller PR, Sauer RT (1996). Role of a peptide tagging system in degradation of proteins synthesized from damaged messenger RNA. Science.

[b22] Klausner RD, Rouault TA, Harford JB (1993). Regulating the fate of mRNA: the control of cellular iron metabolism. Cell.

[b23] Komar AA, Lesnik T, Reiss C (1999). Synonymous codon substitutions affect ribosome traffic and protein folding during *in vitro* translation. FEBS Lett.

[b24] Kuhn LC, Hentze MW (1992). Coordination of cellular iron metabolism by post-transcriptional gene regulation. J Inorg Biochem.

[b25] Leskiw BK, Lawlor EJ, Fernandez-Abalos JM, Chater KF (1991). TTA codons in some genes prevent their expression in a class of developmental, antibiotic-negative, *Streptomyces* mutants. Proc Natl Acad Sci USA.

[b26] Letzring DP, Dean KM, Grayhack EJ (2010). Control of translation efficiency in yeast by codon–anticodon interactions. RNA.

[b27] Li W, Wu J, Tao W, Zhao C, Wang Y, He X (2007). A genetic and bioinformatic analysis of *Streptomyces coelicolor* genes containing TTA codons, possible targets for regulation by a developmentally significant tRNA. FEMS Microbiol Lett.

[b28] Liu H, Styles CA, Fink GR (1996). *Saccharomyces cerevisiae* S288C has a mutation in *FLO8*, a gene required for filamentous growth. Genetics.

[b29] Lorenz MC, Heitman J (1997). Yeast pseudohyphal growth is regulated by GPA2, a G protein alpha homolog. EMBO J.

[b30] Martinez-Anaya C, Dickinson JR, Sudbery PE (2003). In yeast, the pseudohyphal phenotype induced by isoamyl alcohol results from the operation of the morphogenesis checkpoint. J Cell Sci.

[b31] Merrick MJ (1976). A morphological and genetic mapping study of bald colony mutants of *Streptomyces coelicolor*. J Gen Microbiol.

[b32] Murray LE, Rowley N, Dawes IW, Johnston GC, Singer RA (1998). A yeast glutamine tRNA signals nitrogen status for regulation of dimorphic growth and sporulation. Proc Natl Acad Sci USA.

[b33] Oliveira CC, McCarthy JE (1995). The relationship between eukaryotic translation and mRNA stability. A short upstream open reading frame strongly inhibits translational initiation and greatly accelerates mRNA degradation in the yeast *Saccharomyces cerevisiae*. J Biol Chem.

[b34] Oliveira CC, Goossen B, Zanchin NI, McCarthy JE, Hentze MW, Stripecke R (1993). Translational repression by the human iron-regulatory factor (IRF) in *Saccharomyces cerevisiae*. Nucleic Acids Res.

[b35] Pan X, Heitman J (1999). Cyclic AMP-dependent protein kinase regulates pseudohyphal differentiation in *Saccharomyces cerevisiae*. Mol Cell Biol.

[b36] Percudani R, Pavesi A, Ottonello S (1997). Transfer RNA gene redundancy and translational selection in *Saccharomyces cerevisiae*. J Mol Biol.

[b37] Pure GA, Robinson GW, Naumovski L, Friedberg EC (1985). Partial suppression of an ochre mutation in *Saccharomyces cerevisiae* by multicopy plasmids containing a normal yeast tRNAGln gene. J Mol Biol.

[b38] Rato C, Amirova SR, Bates DG, Stansfield I, Wallace HM (2011). Translational recoding as a feedback controller: systems approaches reveal polyamine-specific effects on the antizyme ribosomal frameshift. Nucleic Acids Res.

[b39] Rosenberg AH, Goldman E, Dunn JJ, Studier FW, Zubay G (1993). Effects of consecutive AGG codons on translation in *Escherichia coli*, demonstrated with a versatile codon test system. J Bacteriol.

[b40] Sambrook J, Russell DW (2001). Molecular Cloning: A Laboratory Manual.

[b41] Santos MA, Perreau VM, Tuite MF (1996). Transfer RNA structural change is a key element in the reassignment of the CUG codon in *Candida albicans*. EMBO J.

[b42] Schroder M, Chang JS, Kaufman RJ (2000). The unfolded protein response represses nitrogen-starvation induced developmental differentiation in yeast. Genes Dev.

[b43] Schultz DW, Yarus M (1994). tRNA structure and ribosomal function. I. tRNA nucleotide 27–43 mutations enhance first position wobble. J Mol Biol.

[b44] Sharp PM, Li WH (1987). The codon adaptation index – a measure of directional synonymous codon usage bias, and its potential applications. Nucleic Acids Res.

[b45] Takano E, Tao M, Long F, Bibb MJ, Wang L, Li W (2003). A rare leucine codon in *adpA* is implicated in the morphological defect of *bldA* mutants of *Streptomyces coelicolor*. Mol Microbiol.

[b46] Toda T, Uno I, Ishikawa T, Powers S, Kataoka T, Broek D (1985). In yeast, RAS proteins are controlling elements of adenylate cyclase. Cell.

[b47] Tuller T, Carmi A, Vestsigian K, Navon S, Dorfan Y, Zaborske J (2010). An evolutionarily conserved mechanism for controlling the efficiency of protein translation. Cell.

[b48] Varshney U, Lee CP, RajBhandary UL (1991). Direct analysis of aminoacylation levels of tRNAs *in vivo*. Application to studying recognition of *Escherichia coli* initiator tRNA mutants by glutaminyl-tRNA synthetase. J Biol Chem.

[b49] Vega Laso MR, Zhu D, Sagliocco F, Brown AJ, Tuite MF, McCarthy JE (1993). Inhibition of translational initiation in the yeast *Saccharomyces cerevisiae* as a function of the stability and position of hairpin structures in the mRNA leader. J Biol Chem.

[b50] Wach A, Brachat A, Pohlmann R, Philippsen P (1994). New heterologous modules for classical or PCR-based gene disruptions in *Saccharomyces cerevisiae*. Yeast.

[b51] Weiss WA, Edelman I, Culbertson MR, Friedberg EC (1987). Physiological levels of normal tRNA(CAGGln) can effect partial suppression of amber mutations in the yeast *Saccharomyces cerevisiae*. Proc Natl Acad Sci USA.

[b52] Williams I, Richardson J, Starkey A, Stansfield I (2004). Genome-wide prediction of stop codon readthrough during translation in the yeast *Saccharomyces cerevisiae*. Nucleic Acids Res.

