# Supplementary material for: A yeast tRNA mutant that causes pseudohyphal growth exhibits reduced rates of CAG codon translation
Source: Mol Microbiol. 2012 Dec 4;87(2):284–300. doi: 10.1111/mmi.12096 (PMC3664417; doi:10.1111/mmi.12096)

Table S1: Oligonucleotides used in this study (5'-3')

sup70-S1	gcc caa agt cat gag gag agc ttc tac tat aaa cct cac tcg tac gct gca ggt cga c
sup70-S2	aaa ggc acg tga gaa agt gcc ata cca tta gcc gaa tac gat cga tga att cga gct cg
preS1	gct ttg cat tct aat ccc agt tca ag
postS2	cta cca act ggg att tgg cgt cac atc
sup70-S3	gtc aac gcg gcc gcc ttc tac tat aaa cct cac tc
sup70-S4	gtc aac gcg gcc gct tct tcg ata tct ctg gta tg
pRS-forward	gcg cgt aat acg act cac
pRS-reverse	cac ttt atg ctt ccg gct c
atRNA-std_fw2	tat cga taa gct tga tat cga att cct gca gcc cgg ggg atc cac tag ttc cat aaa acc gga agt ttt agt gta cac taa caa cag aag aaa aag gtc cta tag tgt agt gg
atRNA-std_rv2	ccc tca cta aag gga aca aaa gct gga gct cca ccg cgg tgg cgg ccg ctt caa aaa aaa aaa atg atg gtt taa att tcg taa aat acg aaa aat gaa ggg aag gtc cca ccc gga ttc g
sup70-65c	ggt cct ata gtg tag tgg tta tca ctt tcg att ctg att cga aca acc cca gtt cga atc cgg gtg gga cct
sup70-65inv	ggt cct ata gtg tag tgg tta tca ctt tcg gtt ctg att cga aca acc cca gtt cga atc cgg gtg gga cct
atRNA-7033_fw	tat cga taa gct tga tat cga att cct gca gcc cgg ggg atc cac tag ttc cat aaa acc gga agt ttt agt gta cac taa caa cag aag aaa aag gtc tta tag tgt agt gg
atRNA-7033_rv	ccc tca cta aag gga aca aaa gct gga gct cca ccg cgg tgg cgg ccg ctt caa aaa aaa aaa atg atg gtt taa att tcg taa aat acg aaa aat gaa ggg aag gtc tca ccc gga ttc g
sup70-33c	ggt ctt ata gtg tag tgg tta tca ctt tcg gtt ctg atc cga aca acc cca gtt cga atc cgg gtg aga cct
sup70-33inv	ggt ctt ata gtg tag tgg tta tca ctt tcg gtt ctg atc cga aca acc cca gtt cga atc cgg gtg gga cct
sup70-U33C	ggt cct ata gtg tag tgg tta tca ctt tcg gtc ctg atc cga aca acc cca gtt cga atc cgg gtg gga cct
sup70-U33A	ggt cct ata gtg tag tgg tta tca ctt tcg gta ctg atc cga aca acc cca gtt cga atc cgg gtg gga cct
sup70-U33G	ggt cct ata gtg tag tgg tta tca ctt tcg gtg ctg atc cga aca acc cca gtt cga atc cgg gtg gga cct
Luc-R	ctg gca tgc gag aat ctg
5CAG-luc-F	gga tcc caa tta tct act taa gaa cac aaa act cga gaa cat atg tca cag cag cag cag cag aga gag gat gcc aaa aac ata aag aaa ggc c
10CAG-luc-F	gga tcc caa tta tct act taa gaa cac aaa act cga gaa cat atg tca cag cag cag cag cag aga tca cag cag cag cag cag aga gag gat gcc aaa aac ata aag aaa ggc c
5CAA-luc-F	gga tcc caa tta tct act taa gaa cac aaa act cga gaa cat atg tca caa caa caa caa caa aga gag gat gcc aaa aac ata aag aaa ggc c
10CAA-luc-F	gga tcc caa tta tct act taa gaa cac aaa act cga gaa cat atg tca caa caa caa caa caa aga tca caa caa caa caa caa aga gag gat gcc aaa aac ata aag aaa ggc c
FLuc-f	gaa gcg acc aac gcc ttg att gac aag gat gga tgg cta c
FLuc-r	cac aaa caca ac tcc tcc g
ACT1-f	gat ctg gca tca tac ctt cta c
ACT1-r	gta aca cca tca ccg gaa tcc

<u>Figure S1</u> Example derivation of the chain formation index for quantifying pseudohyphal growth

Panel A; an example experimental measurement of chain formation in a wild-type strain (open bars), and a counterpart *sup70* chain forming allele (closed bars), showing significantly greater numbers of longer chains formed in the mutant cell culture

Panel B; The values p_L were calculated for each culture, for chain lengths >3 cells comprising the total percentage of the population contained within chain length bins ≥ *L*. Thus p_L where *L* = 7 is calculated by adding together percentage of culture with chain lengths 7, 8, 9, 10 and >10, the last five bins of Panel A, and p_L where *L* = 1 is simply equal to the entire population (100% of the population trivially has chain length larger than or equal to 1). The cumulative sum S_C is defined as Σp_L. Calculating S_C effectively integrates the area under the curve defined by all the bars. It can be seen from the graph that this summed bar area is greater for the mutant (area of black bars for chain lengths of 4 or greater = 235 summed units) than the wild-type (area of open bars for chain lengths of 4 or greater = 26 summed units). The chain-forming index CFI is simply a ratio of mutant S_C to wildtype S_C (235/26= 9).

A

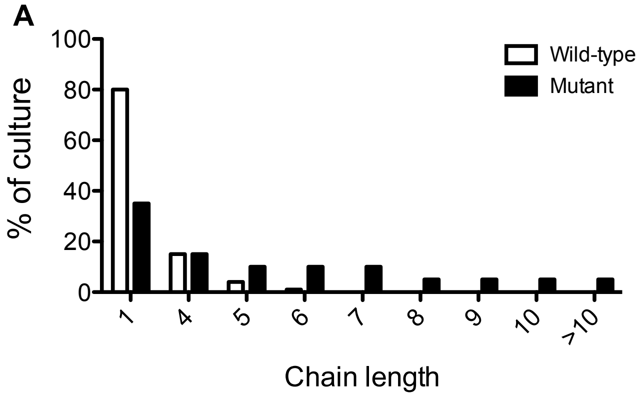

B

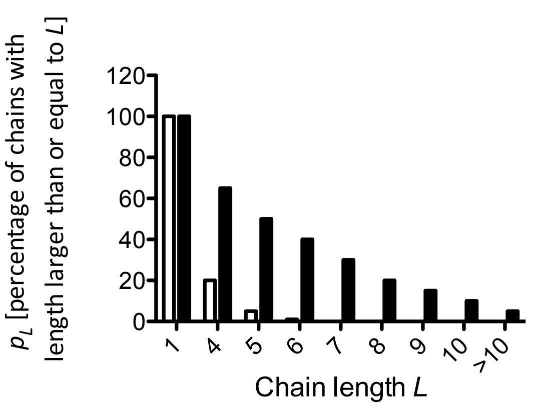

<u>Figure S2</u> $tRNA_{CUG}^{Gln}$ levels are also depleted in *sup70-33* homozygous mutants

We sought independent verification that pseudohyphal mutant alleles of the *SUP70* gene encoded tRNAs that were in some way less abundantly expressed, perhaps because of reduced stability. Mutant strains LMDWU (wild-type *SUP70/SUP70*) and two strains carrying alleles known to be pseudohyphal (Murray *et al*., 1998), LMD651U (*sup70-65/sup70-65*) and LMD6533LU (*sup70-33/sup70-33*) were grown in YPD medium and total tRNAs extracted. tRNAs were resolved on denaturing acrylamide gels, Northern blotted and probed for $tRNA_{CUG}^{Gln}$, and also for $tRNA_{UUG}^{Gln}$ and $tRNA^{His}$ as loading controls Bound probe was quantified using a phosphoimager, and loadings normalised using the $tRNA^{His}$ abundances. The bar chart shows the abundance of $tRNA_{CUG}^{Gln}$ in each of the strains, normalised to the value in the wild-type strain.

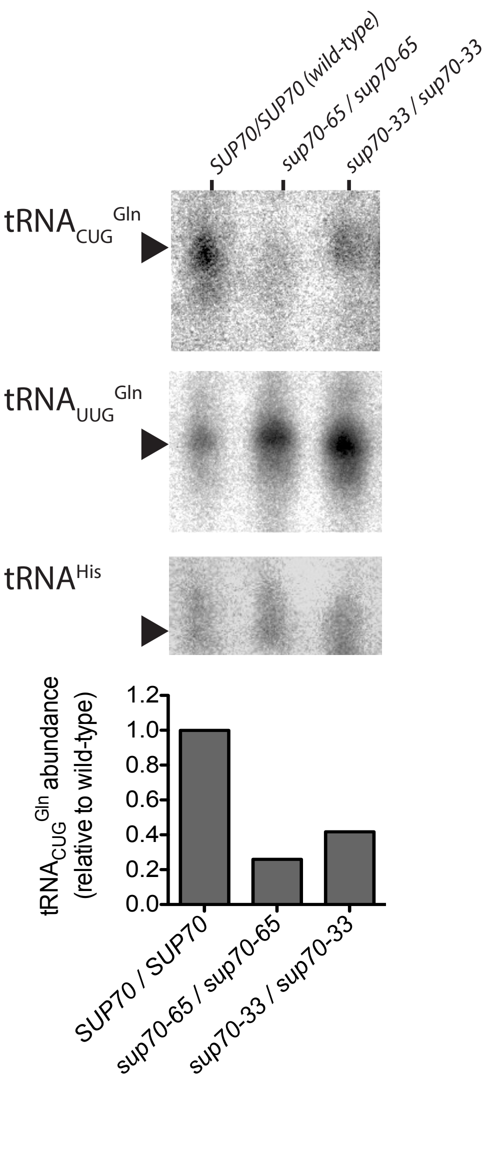

Supplement: Supplementary file 1 [file mmi0087-0284-SD1.pdf]
